# Does Dark-Spot Syndrome Experimentally Transmit among Caribbean Corals?

**DOI:** 10.1371/journal.pone.0147493

**Published:** 2016-01-20

**Authors:** Carly J. Randall, Adán G. Jordán-Garza, Erinn M. Muller, Robert van Woesik

**Affiliations:** 1 Department of Biological Sciences, Florida Institute of Technology, Melbourne, Florida, United States of America; 2 Mote Marine Laboratory, Sarasota, Florida, United States of America; U.S. Geological Survey, UNITED STATES

## Abstract

Over the last half-century, coral diseases have contributed to the rapid decline of coral populations throughout the Caribbean region. Some coral diseases appear to be potentially infectious, yet little is known about their modes of transmission. This study experimentally tested whether dark-spot syndrome on *Siderastrea siderea* was directly or indirectly transmissible to neighboring coral colonies. We also tested whether open wounds were necessary to facilitate disease transmission. At the completion of the experiments, we sampled bacterial communities on diseased, exposed, and healthy coral colonies to determine whether bacterial pathogens had transmitted to the susceptible colonies. We saw no evidence of either direct or waterborne transmission of dark-spot syndrome, and corals that received lesions by direct contact with diseased tissue, healed and showed no signs of infection. There were no significant differences among bacterial communities on healthy, exposed, and diseased colonies, although nine individual ribotypes were significantly higher in diseased corals compared with healthy and exposed corals, indicating a lack of transmission. Although our experiments do not fully refute the possibility that dark-spot syndrome is infectious and transmissible, our results suggest that *in situ* macroscopic signs of dark-spot syndrome are not always contagious.

## Introduction

Over the last half-century, marine diseases have contributed to the wide-spread decline of Caribbean corals [1–4]. Despite decades of research, the etiologies of most coral diseases are poorly understood. Several coral diseases are known to be infectious, and are associated with pathogens such as bacteria, protists, fungi, or with a consortium of microbes [5–16]. Yet the degree to which infectious diseases are contagious is highly variable [17], and few studies have experimentally tested the potential transmission of several common coral diseases in the Caribbean. Understanding the dynamics of coral-disease transmission is necessary to accurately predict future outbreaks of coral diseases, and to potentially mitigate the spread of those diseases.

Infectious diseases are caused by the invasion of a host by a pathogenic agent [17–18]. In veterinary epidemiology, there are two types of infectious agents: exogenous pathogens and endogenous pathogens [17]. Exogenous pathogens are present in the external environment, and are acquired by exposure to a carrier organism, to a non-biological vector, or to an infected host. These pathogens cause diseases that have clear clinical signs and have distinct pathological lesions [17]. By contrast, endogenous pathogens often are present in healthy hosts, but cause disease only when the host becomes stressed. For most coral diseases, it is unknown whether the diseases are caused by exogenous or endogenous pathogens, or if instead, they are the result of non-infectious diseases [19].

Addressing the question of coral-disease transmission has proved difficult for many reasons, including the following: firstly, pathogens for many diseases are unknown or are inconsistently diagnosed [20–22]. Secondly, the diagnosis of infection is based on the subjective identification of macroscopic signs, potentially of different origins [21, 23]. Thirdly, complex interactions among the pathogens, the host corals, and the environment may exist, which could result in successful transmission of a disease under one set of environmental conditions, and in unsuccessful transmission of a disease under another set of environmental conditions [24]. Fourthly, unknown and potentially complex stages of a disease can exist whereby transmission succeeds when the disease is in one stage, and fails when the disease is in another stage [25]. Fifthly, there is the potential existence of vectors, or carriers, in which disease signs do not manifest [26]. Despite these difficulties, examining coral-disease transmission is necessary for elucidating disease etiology.

In this study we define contagious diseases as those which are transmissible through direct physical contact between corals, or which are transmissible indirectly through a vector [27]. Given that corals are sessile and that Caribbean reefs currently support low coral cover [28], physical contact between corals is limited, and it is likely, therefore, that direct transmission of contemporary coral diseases is uncommon [29]. It is more likely that if coral diseases are being transmitted throughout the Caribbean region, the transmission would be indirect, either via a waterborne pathogen or through a reef-associated vector.

Dark-spot syndrome is one of the most prevalent and ubiquitous coral diseases in the Caribbean. Although this disease is thought to be potentially infectious and transmissible, dark-spot transmission has yet to be experimentally demonstrated [30–37]. Dark-spot syndrome affects primarily *Siderastrea siderea*, *O*. *faveolata*, and *Stephanocoenia intersepta*, although it has been observed on at least seventeen species of scleractinian corals in the Caribbean [30, 34, 38, 39], and some researchers have argued that the extent of dark-spot syndrome is an important indicator of overall reef health [33, 40]. A causative agent of dark-spot syndrome has not been identified, although several infectious agents have been proposed, including bacteria from the genera *Vibrio* [32], *Corynebacterium*, *Photobacterium*, and *Acinetobacter*, [36], bacteria from the family *Parvularculaceae* [36], fungi resembling *Aspergillus* spp. [35] and *Rhytisma* spp. [36], and cyanobacteria resembling *Oscillatoria* spp. [36]. A study recently published by Kellogg et al. [37] found no significant distinctions between bacterial communities in healthy and dark-spot affected *S*. *siderea* from St. John, USVI and the Dry Tortugas, suggesting that bacterial pathogens were not the causative agents of the disease. Some assessments suggest that dark-spot syndrome affects primarily the symbiotic zooxanthellae within the coral tissue, causing impairment of mitosis, and a reduction in the density of symbionts [31]. Yet, field studies have found that dark-spot syndrome on corals can result from the physical abrasion of the coral tissue, and that disease signs do not always cause tissue mortality [33, 41]. This has led some researchers to suggest that dark-spot syndrome is a stress response, rather than an infectious disease [19, 33].

The objectives of the present study were to: (1) test direct-contact transmission and waterborne transmission of dark-spot syndrome on *Siderastrea siderea*; and (2) compare the bacterial communities on diseased, disease-exposed, and healthy *Siderastrea siderea* colonies at the completion of the experiment to examine whether potential bacterial pathogens transmitted to the exposed colonies.

## Materials and Methods

### Ethics statement

Coral collections took place under a permit issued by the Florida Keys National Marine Sanctuary numbered FKNMS-2013-086. No ethical approval was required for the laboratory research described in this study.

### Coral collection and experimental system

**Fig 1:**
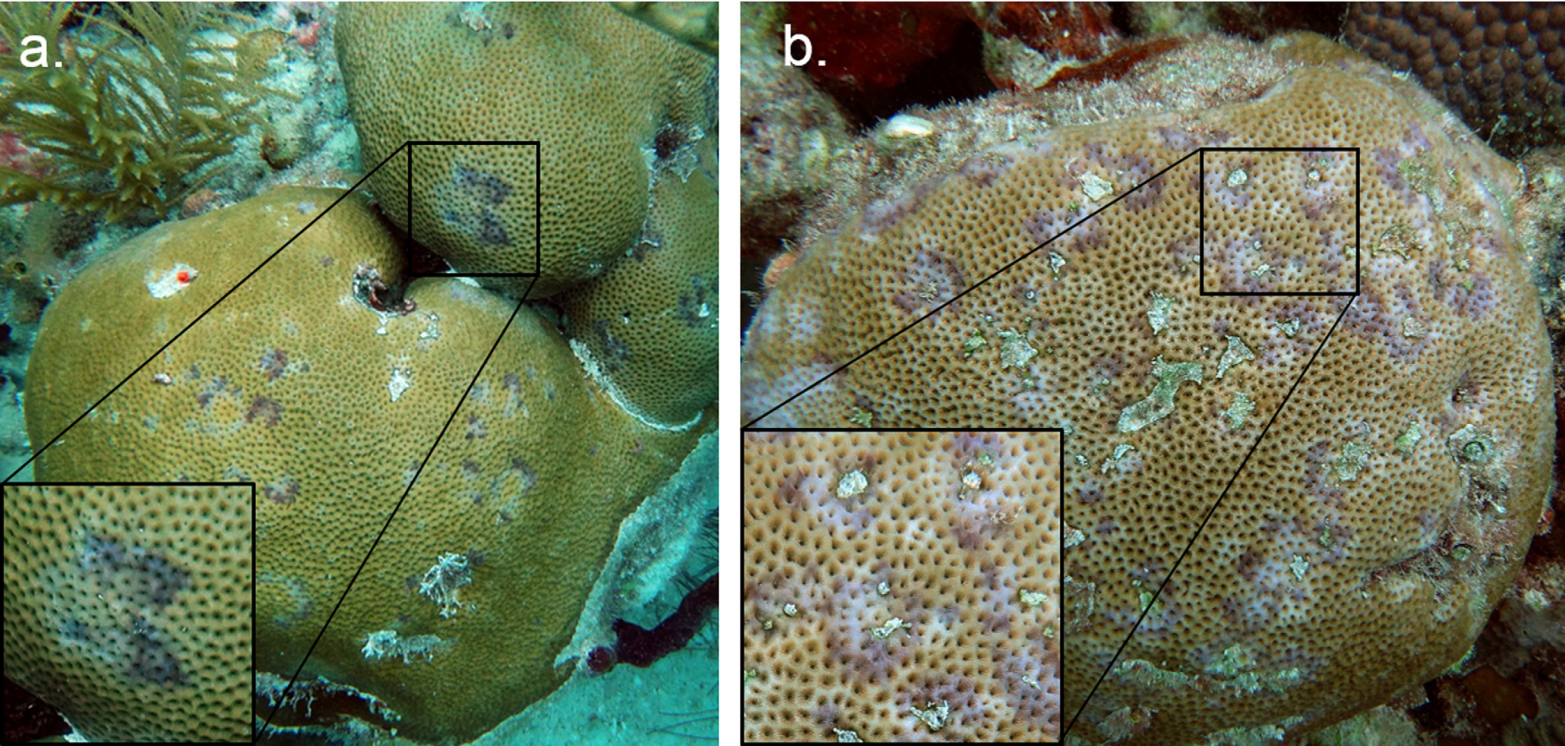
Donor coral colonies. Representative donor colonies (a and b) sampled for use in experiments testing the transmission of dark-spot syndrome on *Siderastrea siderea*. Inset images show disease lesions in detail.

A series of laboratory experiments were conducted on *Siderastrea siderea* to determine whether dark-spot syndrome is transmissible. *S*. *siderea* was selected for the transmission experiments because it is one of the most common hosts of dark-spot syndrome [30, 31, 38, 39]. Diseased corals were selected based on their macroscopic clinical signs and on the presence of pathological lesions. As per Work and Aeby’s [42] guidelines, the case definition of dark-spot syndrome was: multifocal, circular or irregularly shaped purple to brown colored lesions that are centrally or peripherally located, and range from mild (1–20%) to moderate (21–50%) extent on individual colonies. Some lesions were associated with centrally located tissue loss, and the lesions most closely resembled Type I and Type IV lesions as defined by Borger [33] (Fig 1).

Using a hammer and chisel, fragments of *S*. *siderea* colonies were collected from Wonderland Reef in the lower Florida Keys (N 24.56028°, W 81.50127°) in July 2013. Eight diseased and four apparently healthy *S*. *siderea* fragments were collected at a depth of 6–7 m. The number of diseased colonies available for sampling was limited by the number of diseased colonies at the site of collection (which was approximately 200 m by 100 m). In addition, ten healthy colonies (20–90 cm^2^) were collected from the inshore coral nursery in Key West, Florida, to limit damage to corals on Wonderland Reef. All wounds produced by sampling were covered by an epoxy resin to minimize further damage to the corals. All fragments were collected by divers wearing surgical gloves, and healthy fragments were collected prior to diseased fragments, to prevent the potential exposure of healthy fragments to disease during the collection process. All corals were individually packaged in Ziploc® bags with seawater and transported in separate coolers, for healthy and diseased corals, to Mote Marine Laboratory in Summerland Key. Immediately following collection and transportation, corals were fragmented, with a bleach-rinsed (25% bleach solution) wet-tile saw, into ~16 cm^2^ colonies, mounted on 2.5 cm wide by 2.0 cm tall polyvinyl chloride (PVC) bases with Plastilina® modeling clay, and placed into randomly assigned treatment tanks. All corals were handled with surgical gloves, and all equipment was rinsed with a 25% bleach solution in-between the handling of each coral fragment.

The outdoor experimental system was supplied with flow-through seawater from Mote Marine Laboratory’s well-water system [43]. Seawater was off-gassed in a head-tank to maintain pH at 7.9–8.1, and was mechanically filtered with a sand-particle filter. Seawater entering the flow-through system was ultraviolet-light (UV) sterilized with an 18W Coralife® Turbotwist UV Sterilizer to ensure that the supply of water was free of pathogens. The system was covered with shade cloth to match the irradiance levels in the aquaria as closely as possible with the irradiance levels that were measured at the site of coral collection (58 μmol quanta m^-2^ s^-1^ at approximately 10:50 am). All experiments were conducted between 10 July and 14 August 2013 at Mote Marine Laboratory in Summerland Key, Florida.

### Experiment 1: Waterborne transmission

**Fig 2:**
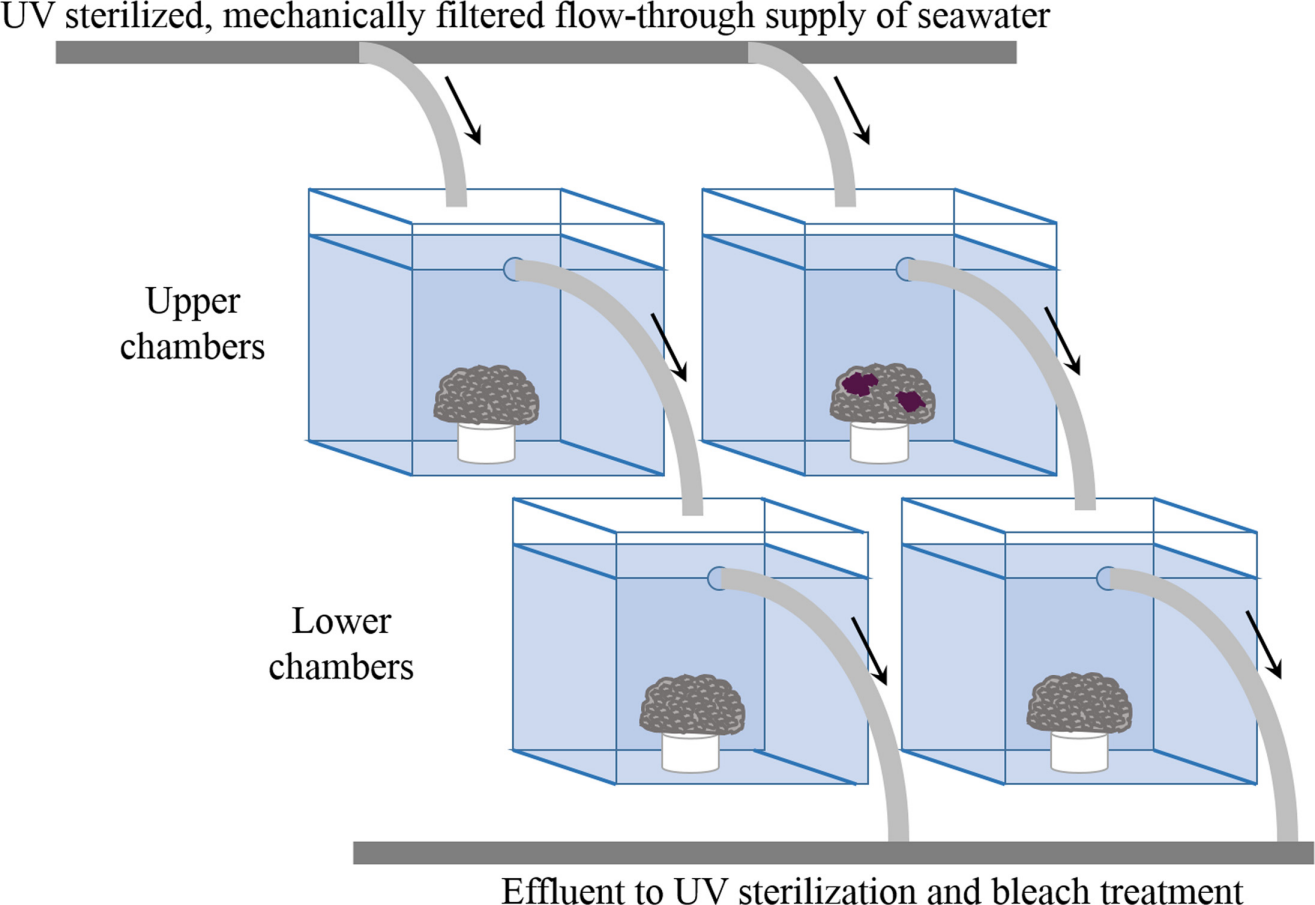
Cascading experimental design. The design had unidirectional water flow (arrows) from upper chambers (2.7 L each) to lower chambers (2.7 L each), which allowed the healthy corals in the lower chambers to be exposed to water from the upper-chambers housing diseased corals. All upper chambers were supplied with UV sterilized and mechanically filtered seawater, and all effluent water was treated with UV sterilization and bleach, before allowing the water to drain into a nearby canal.

**Fig 3:**
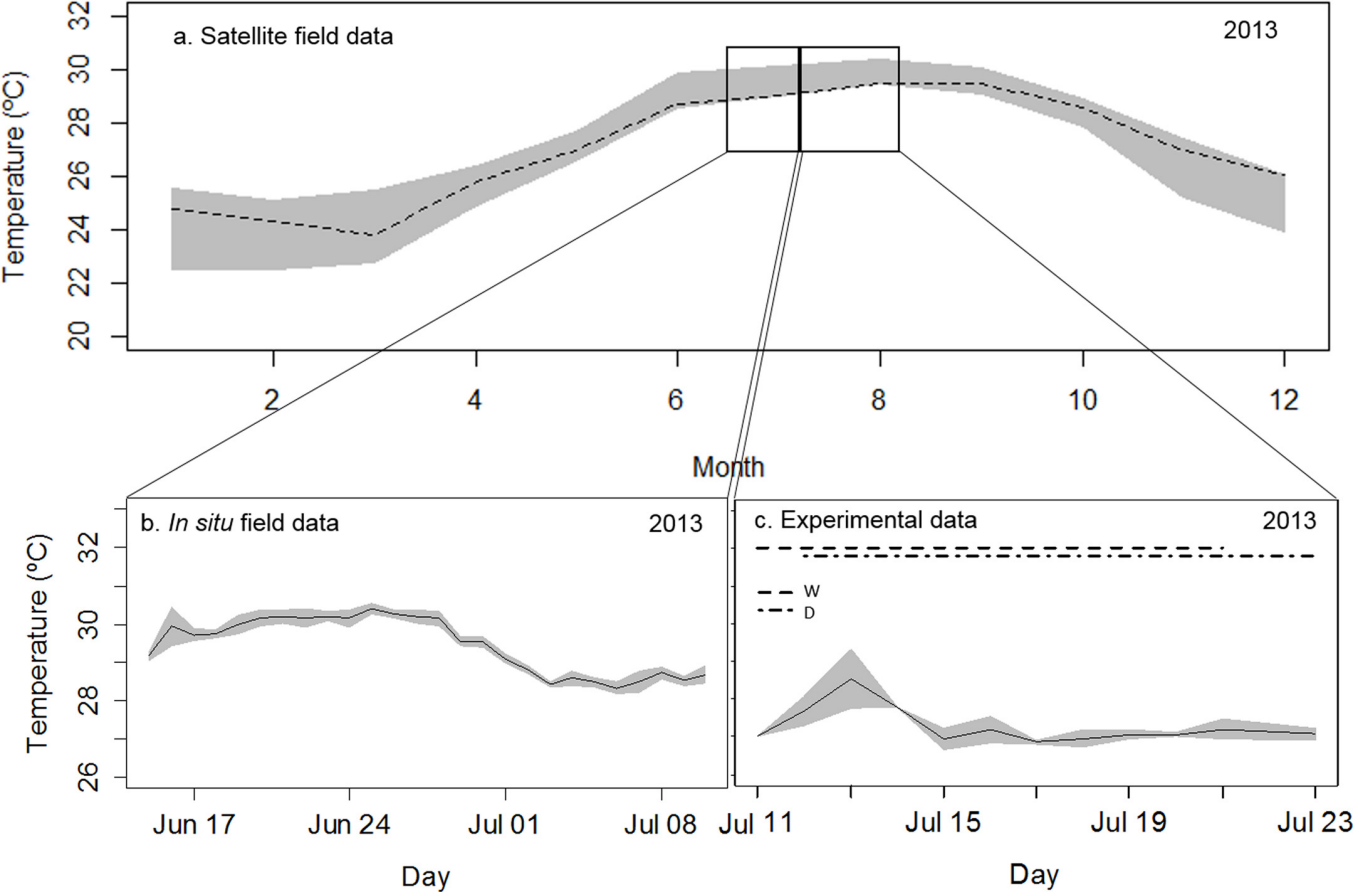
Thermal history of the corals tested in a series of laboratory-based transmission experiments. **a**. Mean monthly sea-surface temperature (dotted line) and the minimum and maximum of the 10-year monthly mean (gray range) obtained from HadI sea-surface temperature records for the site of collection [44]. **b**. Mean daily sea-surface temperature from a Hobo® data logger deployed near the site of collection (data logged every 15 minutes) and the standard deviation around the mean (gray range). **c**. Mean daily experimental temperatures measured with a standard laboratory thermometer for the duration of the transmission experiments. Gray range = standard deviation around the mean; W = waterborne transmission, D = direct-contact transmission.

The system for the waterborne-transmission experiments consisted of sixty (previously unused) 2.7 L acrylic aquaria, set-up in a cascading design (Fig 2). This design allowed for unidirectional flow to test waterborne-disease transmission by exposing corals in the ‘lower chambers’ to water that had been previously circulated in the ‘upper chambers’ housing diseased corals. Flow rates in all aquaria were measured and calibrated daily. The flow rates averaged 28 ± 4 L hr^-1^ (an average of 2 cm sec^-1^ with 11 volume turnovers per hour, and a residence time of 6 minutes). Water temperatures were measured daily with a standard laboratory thermometer and averaged 27.4°C (± 0.6°C SD; Fig 3).

Four treatments were included in the experimental design: (i) transmission from a diseased-coral colony to a healthy-coral colony (experimental); (ii) transmission from a healthy-coral colony to a healthy-coral colony (control 1); (iii) transmission from a rubble fragment to a healthy-coral colony (control 2); and (iv) transmission from an empty chamber to a healthy-coral colony (control 3). Seven replicates of each treatment pair were tested. The waterborne-transmission experiment was run for 11 days, after which the experimental corals were placed in a holding tank and monitored for an additional 17 days (for a total of 28 days). All coral colonies were monitored for signs of disease and photographed daily.

To compare the photochemical yields of colonies within each treatment, pulse amplitude modulated (PAM) fluorometry measurements were taken three hours after sunset (i.e., three-hour dark-acclimated) on Days 6 and 9 of the experiment. Two technical-replicate measurements were taken on each colony on different locations within the same colony, and were averaged to obtain one measurement of photochemical yield per colony. PAM measurements of the diseased colony were taken directly on tissue with visible lesions. The PAM probe was rinsed with a 10% bleach solution, and then rinsed with sterilized seawater in-between each measurement.

A repeated measures analysis of variance (RM-ANOVA) was used to assess differences in photochemical yields among treatments on the same corals through time. Photochemical-yield data met the assumptions of RM-ANOVA, and statistical analyses were conducted using base R, and the packages ‘car’ [45] and ‘userfriendlyscience’ [46] in the statistical program R [47].

### Experiment 2: Direct-contact transmission

The system for the direct-contact transmission experiment consisted of six (previously unused) 25 L plastic aquaria. All aquaria received flow-through, filtered, and UV-sterilized seawater, as described above. Flow rates to all aquaria were measured and calibrated daily, and averaged 48 ± 6 L hr^-1^ (an average of 2 cm sec^-1^, with a 2-volume turnover per hour, and a residence time of 32 minutes). Water temperature was measured daily using a standard laboratory thermometer and averaged 27.5°C (± 0.8°C SD) (Fig 3).

**Fig 4:**
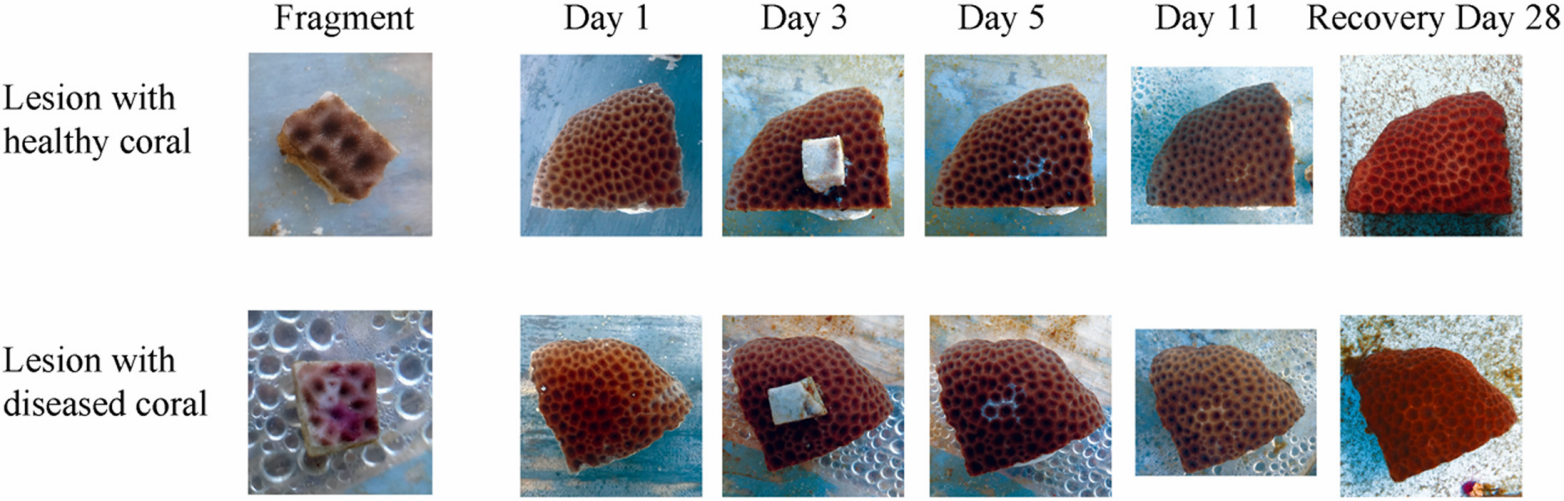
Time-series images of two healthy-coral colonies (~16 cm^2^) of *Siderastrea siderea* in a direct-contact transmission experiment. The first column of images is a close-up of the fragments (~1 cm^2^) that were placed in direct contact with the coral colonies. The first row of images show a healthy-coral colony (~16 cm^2^) in direct contact with a healthy fragment (~1 cm^2^) (control). The second row of images show a healthy-coral colony in direct contact with a fragment with dark spot lesions (~1 cm^2^) (experimental). Note that Day 1 images were taken prior to the addition of the direct-contact coral fragment. Note also the direct-contact lesion scars on *S*. *siderea* on Day 5.

Two treatments were included in the experimental design: (1) direct-contact from a diseased-coral fragment (~1 cm^2^) to a healthy-coral colony (~16 cm^2^) (experimental); and (2) direct-contact from a healthy-coral fragment (~1 cm^2^) to a healthy-coral colony (~16 cm^2^) (control). Three replicates were tested for each treatment. On Day 1, small fragments of *S*. *siderea*, (~1 cm^2^, either diseased or healthy depending on the treatment) were placed directly on the top of healthy *S*. *siderea* colonies (~16 cm^2^), with direct tissue-to-tissue contact (Fig 4). The coral fragments were left in direct contact with the healthy-coral colonies for three days, and were then removed. After a total of five days (i.e., two days following fragment removal), no dark-spot transmission was observed. Therefore, on Day 5, lesions were created on the healthy *S*. *siderea* colonies to test the hypothesis that direct-contact transmission only occurs when a wound is present, allowing for pathogen entry. Lesions were created by forcibly grinding the coral fragments into the healthy-coral colonies, thereby breaking the healthy tissue and damaging the coral skeleton, so that potential pathogens, wherever they may be located in the colony, would be exposed to the healthy coral colonies (Fig 4, Day 5). Coral fragments were left in contact with the injured tissue of the healthy-coral colonies for 13 hours to facilitate potential pathogen transmission, and then removed. The *S*. *siderea* colonies were monitored for six more days (for a total of 11 days) and were photographed daily for the duration of the experiment, after which all *S*. *siderea* colonies were placed in a holding tank and monitored for an additional 17 days (for a total of 28 days).

### Examination of the transmission of potential bacterial pathogens

Immediately following completion of the waterborne-transmission experiments (Experiment 1), three each, of diseased, exposed, and healthy colonies of *S*. *siderea* were randomly selected for bacterial-community analyses, to determine whether potential bacterial pathogens had transmitted to the exposed colonies. The nine coral colonies were placed in individual, sterile whirl-paks at -80°C and then were transported on dry ice to Mote Marine Laboratory in Sarasota, Florida.

Tissue was removed from the skeleton of the preserved-coral colonies using a Paasche® airbrush with 10 mL of sterile seawater. The tissue slurry was collected in a sterile 50 mL Falcon® tube and homogenized using a vortex. The tissue homogenate was then spun down into a pellet using a centrifuge set at 10,000 rpm. The pellet was re-suspended in 2 mL of solution C1 and DNA was extracted using a Powersoil DNA extraction kit (MoBIO Laboratories Inc. Lot #PS14F19). Extracted DNA was then sent to MRDNA Laboratory (www.mrdnalab.com, Shallowater, TX, USA) for Illumina® sequencing (20,000 reads per assay) using the universal bacterial primers 27F/519R with a barcode on the forward primer. The 16S rRNA gene on the V1–V3 hypervariable region was amplified by applying a 30 cycle polymerase chain reaction (PCR) with the HotStarTaq Plus Master Mix Kit (Qiagen, USA). PCR was applied using the following protocol: (1) 94°C for 3 minutes, (2) 28 cycles of: 94°C for 30 seconds, 53°C for 40 seconds, and 72°C for 1 minute, and (3) a final elongation step at 72°C for 5 minutes. After amplification, PCR products were confirmed in 2% agarose gels to determine the success of amplification and the relative intensity of the bands. Multiple samples were pooled together in equal proportions based on their molecular weight and DNA concentrations. Pooled samples were purified using calibrated Ampure® XP beads. Then the pooled and purified PCR product was used to prepare DNA libraries by following the Illumina® TruSeq DNA library preparation protocol. Sequencing was performed using the Illumina® sequencing platform at MR DNA (www.mrdnalab.com, Shallowater, TX, USA) following the manufacturer’s guidelines. Sequence data were processed using a standardized analysis pipeline. Briefly, sequences were initially depleted of barcodes. Then sequences less than 150bp or with ambiguous base calls were removed. Operational taxonomic units (OTUs) were generated, and chimeras were removed using UCHIME [48]. OTUs were defined by clustering at 3% divergence (i.e., showing 97% similarity) using a de novo method. Final OTUs were taxonomically classified using BLASTn against the curated National Center for Biotechnology Information (NCBI) database and the Ribosomal Database Project (RDP).

The relative-percentage contributions of each genus (and higher taxonomic levels) to the bacterial assemblages were calculated for each of the nine coral colonies. Differences in coral-bacterial communities among treatments were tested using a permutational multivariate analysis of variance (PERMANOVA). The results were visualized using non-metric multidimensional scaling plots. Differences in mean bacterial richness among treatments at each taxonomic level were tested using an analysis of variance (ANOVA), or using a Kruskal-Wallis test in cases where the assumptions of ANOVA could not be met through data transformations. Shannon-Weiner diversity indices were calculated for all samples, at each taxonomic level, and differences in diversity among treatments were tested using ANOVA. Differences in the relative abundance of each bacterial taxon among treatments were calculated using a Kruskal-Wallis rank-sum test with a chi-square distribution. Analyses were conducted using the packages ‘vegan’ [49] and ‘ggplot2’ [50] in R [47].

## Results

**Fig 5:**
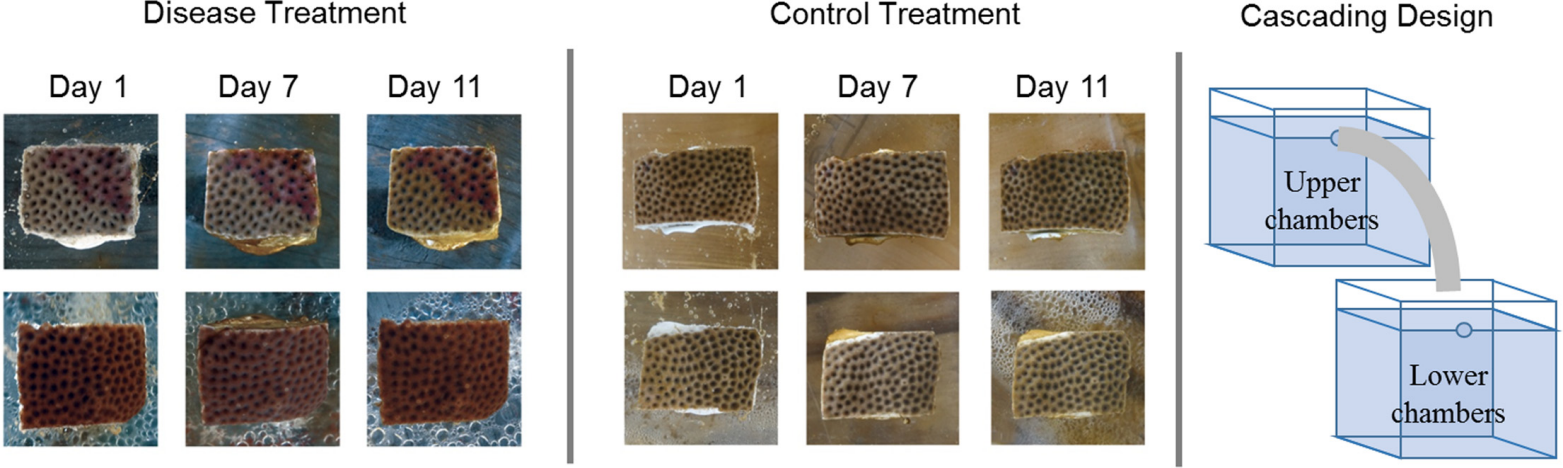
Time-series images of *Siderastrea siderea* from waterborne-transmission experiments. The top row represents diseased or healthy-coral colonies (~16 cm^2^), depending on the treatment, from the upper chambers. The bottom row represents healthy-coral colonies (~16 cm^2^) from the lower chambers.

Dark-spot syndrome did not visually transmit to healthy corals in any experiment in the present study (Figs 4 and 5). There was no apparent transmission to the lesions that were generated by direct contact with diseased fragments, and lesions were completely healed by Day 28 (Fig 4). Analysis of the Illumina® sequencing data revealed no significant differences among the bacterial communities on healthy, exposed, and diseased corals at the completion of the waterborne-transmission experiment (Table 1, S1 and S2 Figs). Furthermore, no significant differences in bacterial richness were detected among treatments (Table 2, S3 Fig), and no significant differences in the relative abundance of six purported pathogens were found among treatments (S1 Table). However, the relative abundance of nine bacterial taxa increased significantly in diseased corals compared with healthy and exposed corals, pointing to potential pathogens, but indicating a lack of transmission (S4 Fig).

**Table 1:** PERMANOVA results of bacterial-community analyses on diseased and healthy corals.

Dark-spot syndrome	df	SS	MS	Pseudo(F)	R^2^	P(perm)
Genus	2	0.35	0.18	1.73	0.37	0.057
Family	2	0.26	0.13	1.83	0.38	0.082
Order	2	0.25	0.13	1.96	0.39	0.069
Class	2	0.13	0.07	1.47	0.33	0.246

Permutational multivariate analyses of variance (PERMANOVA) comparing bacterial communities among healthy, exposed, and diseased-coral colonies (~16 cm^2^) tested in laboratory-based dark spot-transmission experiments on *Siderastrea siderea*. Differences in bacterial communities were examined at each of four taxonomic levels. df = degrees of freedom; SS = sum of squares, MS = mean sum of squares; Pseudo(F) = permuted F-value; P(perm) = permuted p-value.

**Table 2:** Mean bacterial richness on diseased and healthy corals.

Healthy				Exposed			Diseased			ANOVA/Kruskal-Wallis Results			
Taxa	Average	Lower CI	Upper CI	Average	Lower CI	Upper CI	Average	Lower CI	Upper CI	df	F-value	Chi-sq	p-value
Genus	314	262	367	312	247	378	297	236	358	2	0.096	n/a	0.910
Family	145	120	170	144	124	165	139	119	159	2	n/a	1.681	0.432
Order	77	63	91	76	65	86	74	68	79	2	n/a	0.090	0.956
Class	39	31	48	39	33	46	36	32	40	2	0.270	n/a	0.722

Average bacterial richness at four taxonomic levels in healthy, exposed, and diseased-coral colonies (~16 cm^2^) tested in laboratory-based dark-spot syndrome transmission experiments on *Siderastrea siderea*. CI = 95% confidence interval. Results of analysis of variance (ANOVA) (or the non-parametric Kruskal-Wallis test when ANOVA assumptions were not met) comparing bacterial richness among treatments, at each taxonomic level are presented.

### Experiment 1: Waterborne transmission

**Fig 6:**
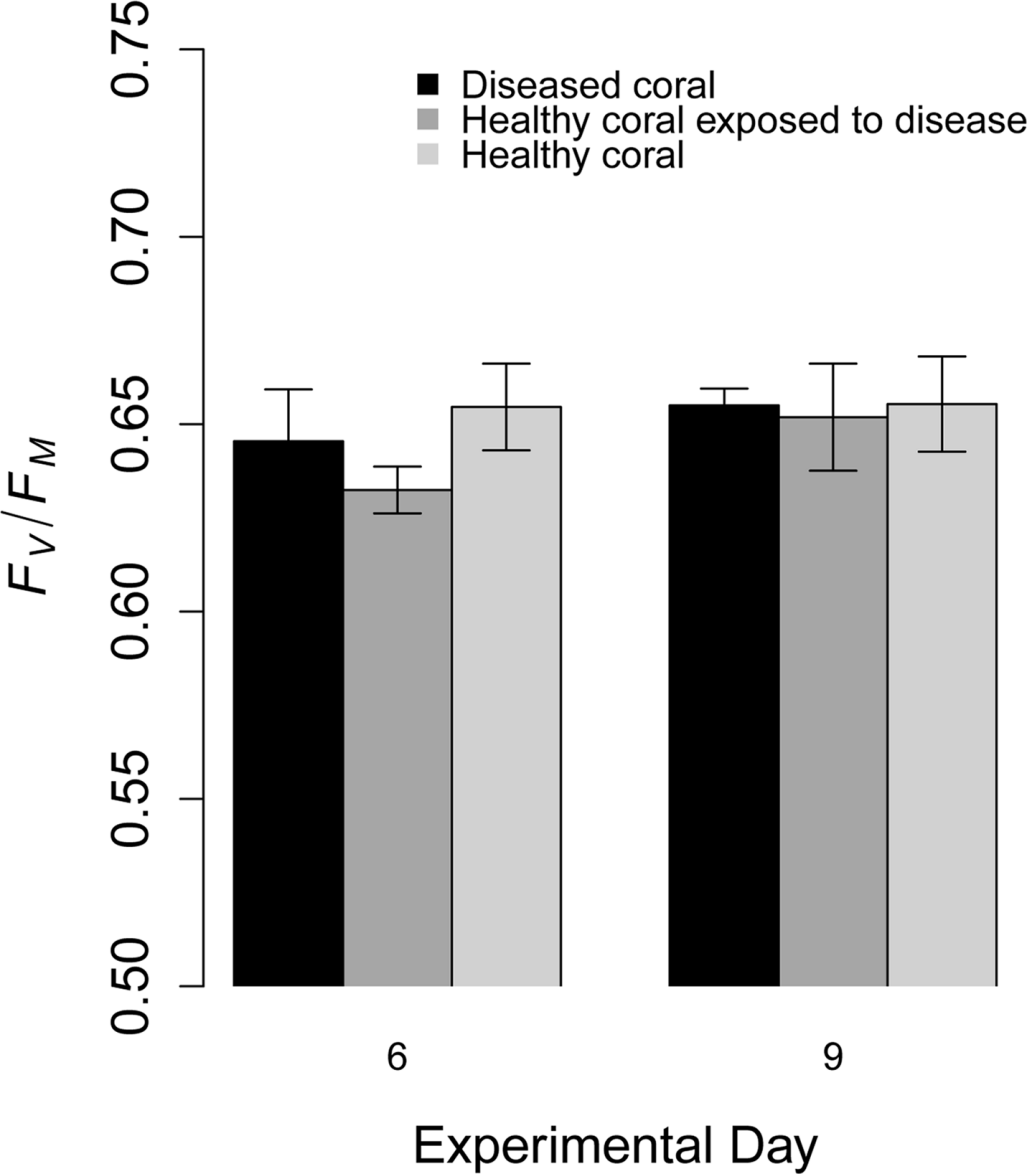
Photochemical yield on corals tested in a waterborne disease-transmission experiment. Mean photochemical yield of 3-hour dark-adapted colonies of *Siderastrea siderea* tested in a dark-spot transmission experiment on two days. Measurements of diseased-coral colonies were taken directly on tissue with dark spots. Where n = 7, and error bars denote standard deviation.

No waterborne transmission of dark-spot syndrome on *S*. *siderea* was observed during the first 11 days (Fig 5), and all exposed corals remained healthy in the holding tanks following the experiments. Throughout the experiment, the size, color, and shape of the dark-spot lesions remained unchanged, indicating that there was no progression or regression of the lesions through time (Fig 5). Furthermore, there were no significant differences in the photochemical yield among treatments or through time (F = 2.17, df = 2, p = 0.125 for treatment, and F = 1.46, df = 1, p = 0.233 for time; Fig 6).

### Experiment 2: Direct-contact transmission

No direct transmission of dark-spot syndrome on *S*. *siderea*, was observed during the first 11 days (Fig 4), and all exposed corals remained healthy in the holding tanks following the experiments (Fig 4). All healthy *S*. *siderea* colonies showed partial recovery from their artificially-induced lesions by Day 11, and did not develop dark spots. Following the recovery period, all healthy-coral colonies had completely healed from their artificially-induced lesions, and no dark-spot syndrome had developed after 28 days (Fig 4).

### Examination of the transmission of potential bacterial pathogens

**Fig 7:**
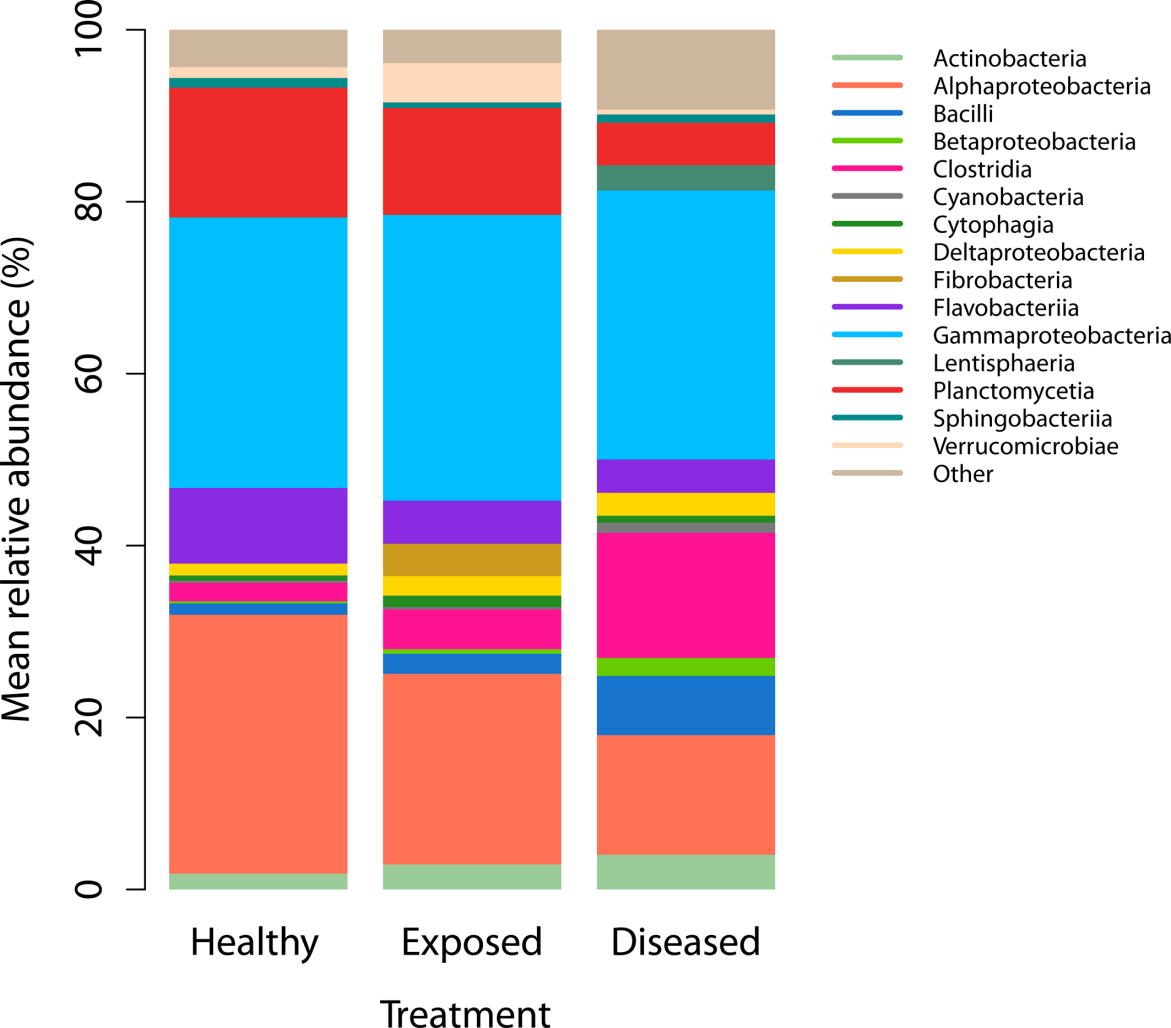
Relative abundance of bacteria in coral tissue. Mean bacterial community structure on healthy, exposed, and dark-spot tissue of *Siderastrea siderea*, including the 10 most abundant taxonomic classes from each experimental condition (totaling 12 classes). None of the most abundant classes were found exclusively in any one treatment.

An average of 98% of the DNA sequenced from each of the nine coral colonies was of Prokaryotic origin, and 4,103 operational taxonomic units (OTUs) that represented 71 different classes were detected across the nine samples of *S*. *siderea* (S2 and S3 Tables). The differences among the bacterial communities on diseased, exposed, and healthy-coral colonies, after allowing for waterborne transmission, were examined at four taxonomic levels (genus to class) using PERMANOVA. No significant differences in the bacterial communities were detected at any taxonomic level (Table 1, Fig 7, S1 and S2 Figs). Furthermore, no significant differences in bacterial richness or species diversity were detected at any taxonomic level (Table 2, S3 Fig), and no significant differences in the relative abundance of six purported pathogen taxa were found among treatments (S1 Table). The same five bacterial classes Alphaproteobacteria, Clostridia, Flavobacteriia, Gammaproteobacteria and Planctomycetia were most abundant in healthy and diseased tissues of *S*. *siderea*. Of the 100 most abundant bacterial genera in dark-spot *S*. *siderea* tissue, which together constituted 94% of the relative abundance, none were absent in healthy *S*. *siderea* tissue. However, taxon-specific Kruskal-Wallis rank-sum tests identified 14 out of 934 taxa, with abundances that differed significantly among treatments. Nine of those taxa were significantly more abundant in diseased corals compared with healthy and exposed corals, and included *Alteromonas* (96% homology), *Aquabacterium* (95% homology), *Arthrobacter* (84% homology), *Bermanella* (95% homology), *Haliscomenobacter* (87% homology), *Litoreibacter* (97% homology), *Oscillatoriales* (92% homology), *Pseudomonas* (96% homology), and *Sorangiineae* (93% homology). Two taxa, *Azospirillum* (90% homology) and *Psychroflexus* (89% homology) were significantly less abundant in diseased corals compared with healthy and exposed corals (S4 Fig).

## Discussion

Dark-spot syndrome was first described in the 1990s [51, 52], yet it remains unknown if, and how, this disease is transmitted through coral populations. The present study tested direct and indirect transmission of this widespread Caribbean-coral disease. We assessed transmission both at the macroscopic level, through the assessment of visual signs, and at the microscopic level, through the analysis of changes in bacterial-community structure, and by testing the physiological integrity of their symbiotic dinoflagellates. Our results demonstrated that dark-spot syndrome was not directly or indirectly transmitted from diseased colonies to healthy-appearing colonies under our experimental conditions.

Dark-spot syndrome is one of the most prevalent disease signs in the Florida Keys [41], in the Caribbean [53, 54], and in the Gulf of Mexico [55]. Currently, there is a debate regarding the etiology of dark-spot syndrome, and whether it is an infectious or a non-infectious disease [19, 31–35]. Our results agree with the recent findings of Kellogg et al. [37], indicating no significant shifts in the overall bacterial communities between diseased and healthy corals. Yet, by comparison, the bacterial communities on *S*. *siderea* that are reported here were strikingly different from those on *S*. *siderea* reported by Kellogg et al. [37]. These results suggest that healthy-coral bacterial communities vary across space and through time (S5 Fig). Alternatively, differences in the bacterial communities detected in the present study and those detected by Kellogg et al. [37] could be the result of the ‘tank effect’ [56, 57], or a result of the use of different detection methods (Illumina® sequencing in the present study versus microarrays in Kellogg et al. [37]). Furthermore, significant increases in a few individual taxa, not detected by the community-level analyses, point to nine potential pathogenic bacterial taxa that warrant further investigation (S4 Fig). Interestingly, none of these taxa, with the exception of *Oscillatoria*, have been previously proposed as pathogens of dark-spot syndrome (S1 Table). However, if indeed any of these taxa are pathogenic, their abundances did not increase significantly in exposed corals, compared with healthy corals, suggesting that they did not transmit in our study (S4 Fig).

It has been suggested that dark-spot syndrome is primarily a disease of the algal symbionts (*Symbiodinium* spp.) that reside within the host-coral tissue [31]. In a study of *S*. *siderea* tissue with dark-spot syndrome, Cervino et al. [31] identified a reduction in the number of symbionts by 56%, a reduction in the mitotic indices of the symbionts, and changes in symbiont physiology. Results from our experiments indicated that there were no significant differences in the photochemical yields of the corals among treatments (Fig 6), suggesting that there were no differences in the ‘health’ of the *Symbiodinium* spp. in the diseased and healthy corals in the experiment. Our results could indicate that the etiology of dark-spot syndrome in our experiments was different from the disease tested by Cervino et al. [31]. Alternatively, it could be that the diseases were in different stages of infectivity, or that a second affliction was causing the reduction in *Symbiodinium* spp. in the corals tested by Cervino et al. [31].

Previous laboratory and field studies of some coral diseases have observed rapid transmission (i.e. < 10 days) from infected to healthy corals [14, 58–62]. By contrast, other coral diseases have taken longer to transmit, or altogether failed to transmit [63–64]. Our results suggest that dark-spot syndrome on *S*. *siderea* is comparably less transmissible than white-sign diseases, ciliate-associated diseases, and black-band disease. Yet, our experiments do not fully refute the possibility that dark-spot syndrome is infectious and transmissible. For example, dark-spot syndrome may need a biological vector that wasn’t present in the experiments, or the disease may take more than 28 days to develop and manifest. It is possible, also, that the disease may be contagious under non-sterile conditions, only during certain stages of the pathogen life-cycle, only under anomalously stressful conditions for the host, or under anomalous conditions that promote pathogen virulence. Additional research examining disease transmission under varying environmental conditions with different potential vectors are needed to addresses these research questions. In conclusion, the results from these transmission experiments indicate that dark-spot syndrome is not a readily transmissible coral disease. While we cannot rule out the possibility of other mechanisms of transmission, these results are a positive step toward understanding the dynamics of a prevalent Caribbean-coral disease.

## Supporting Information

S1 Fig Heatmaps of the relative abundance of bacteria. Heatmaps of the relative abundance of bacteria identified on each sample of healthy, exposed, and diseased coral tissue, tested in waterborne transmission experiments of dark-spot syndrome on *Siderastrea siderea*. Bacteria were examined at the class (a), order (b), family (c), and genus (d) taxonomic levels. Cluster dendrograms are indicated above each heatmap.
(PDF)

S2 Fig Heatmap of the relative abundance of OTUs. Heatmap of the relative abundance of operational taxonomic units (OTUs) identified on each sample of exposed, and diseased coral tissue, tested in waterborne transmission experiments of dark-spot syndrome on *Siderastrea siderea*. A cluster dendrogram is indicated above the heatmap.
(PDF)

S3 Fig Shannon-Weiner diversity indices. Shannon-Weiner diversity of microbes, at six taxonomic levels, in three samples each of healthy (H), exposed (E) and diseased (D) coral tissue, tested in waterborne transmission experiments of dark-spot syndrome on *Siderastrea siderea*.
(PDF)

S4 Fig Mean relative abundance of individual bacterial taxa in diseased, exposed and healthy corals. Only taxa whose relative abundances were significantly different among treatments, as determined by a Kruskal-Wallis rank-sum test using a chi-squared distribution are reported. Error bars denote standard deviation. n = 3.
(PDF)

S5 Fig Comparison of class-level bacterial data from the current study with Kellogg et al. [37]. Cluster dendrogram is based on the relative abundance of class-level bacterial data from healthy and dark-spot affected *Siderastrea siderea* from the Florida Keys in 2013 (present study) compared with the Dry Tortugas and St. John, USVI in 2009 (Kellogg et al. [51]). Sample numbers in black represent apparently-healthy corals, whereas sample numbers in red represent corals with dark-spot syndrome.
(PDF)

S1 Table Results of non-parametric analyses of variance of five purported pathogen taxa. Results of Kruskal-Wallis rank-sum tests using a chi-squared distribution comparing relative abundances of bacteria of six purported pathogenic taxa on diseased, exposed, and healthy corals colonies (~16 cm^2^) at the completion of laboratory-based dark-spot syndrome transmission experiments on *Siderastrea siderea*, where df = degrees of freedom, and n/a indicates that no *Oscillatoria* were identified in any sample.
(DOCX)

S2 Table Absolute abundance of OTUs in each coral sample. Operational taxonomic units (OTUs) and their corresponding classifications and percentage homology for each of nine coral samples of *Siderastrea siderea* tested in waterborne-transmission experiments of dark-spot syndrome. D4U, D13U and D27U were healthy corals; D1L, D3L and D22L were exposed corals; D1U, D3U and D22U were dark-spot affected corals.
(XLSX)

S3 Table Nucleotide sequences for OTUs. Nucleotide sequences for all operational taxonomic units (OTUs) identified on *Siderastrea siderea* corals tested in waterborne transmission experiments of dark-spot syndrome.
(XLSX)
