# Supplementary figures and images for: Does Dark-Spot Syndrome Experimentally Transmit among Caribbean Corals?
Source: PLoS One. 2016 Jan 20;11(1):e0147493. doi: 10.1371/journal.pone.0147493 (PMC4720368; doi:10.1371/journal.pone.0147493)

a.

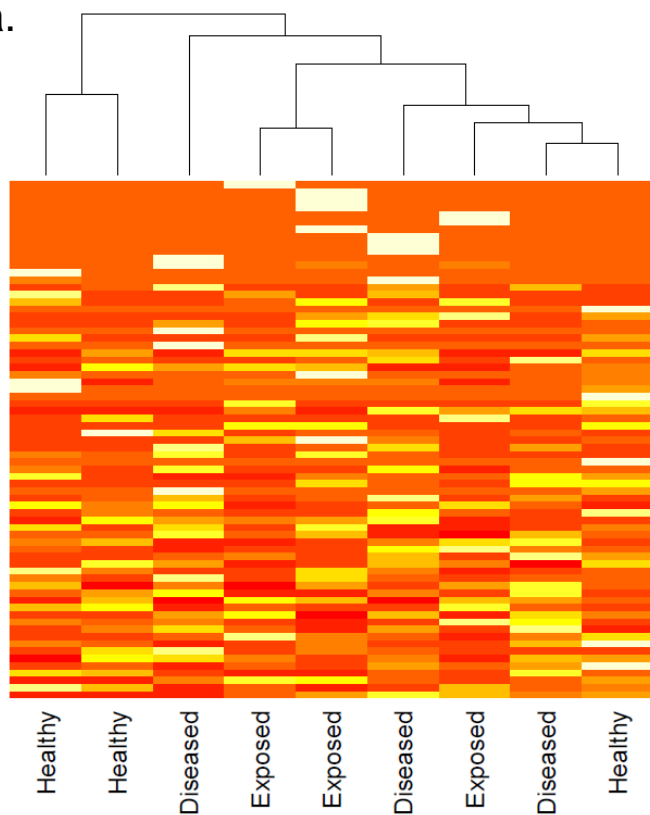

b.

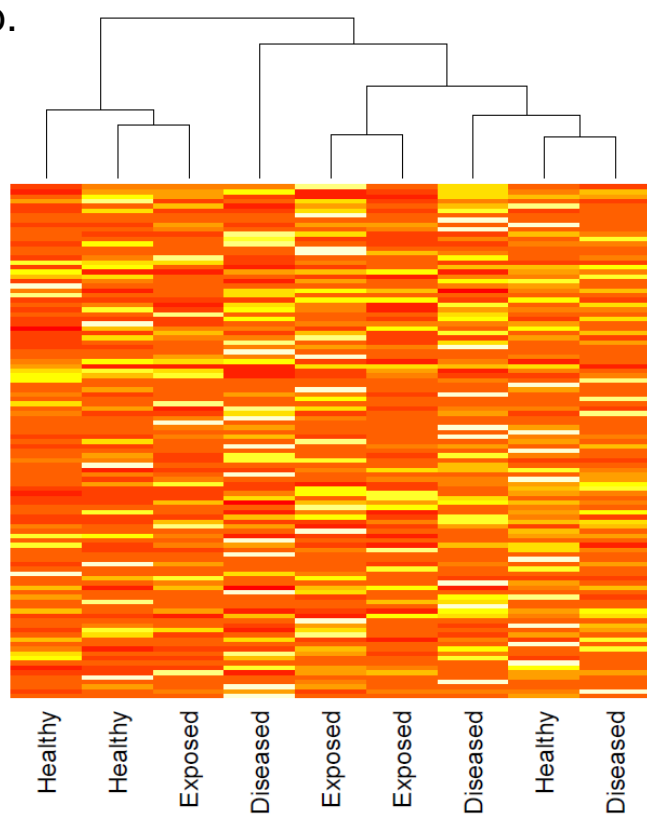

c.

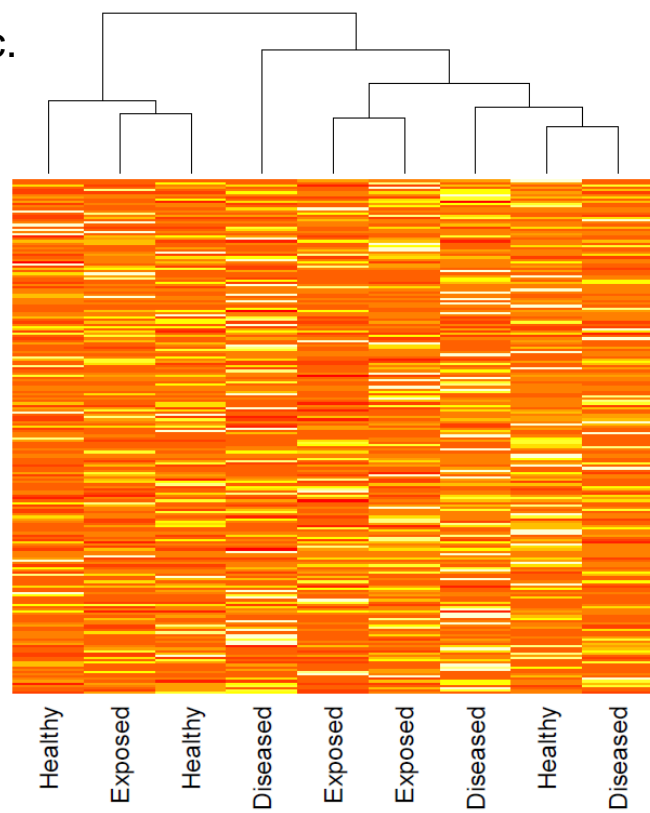

d.

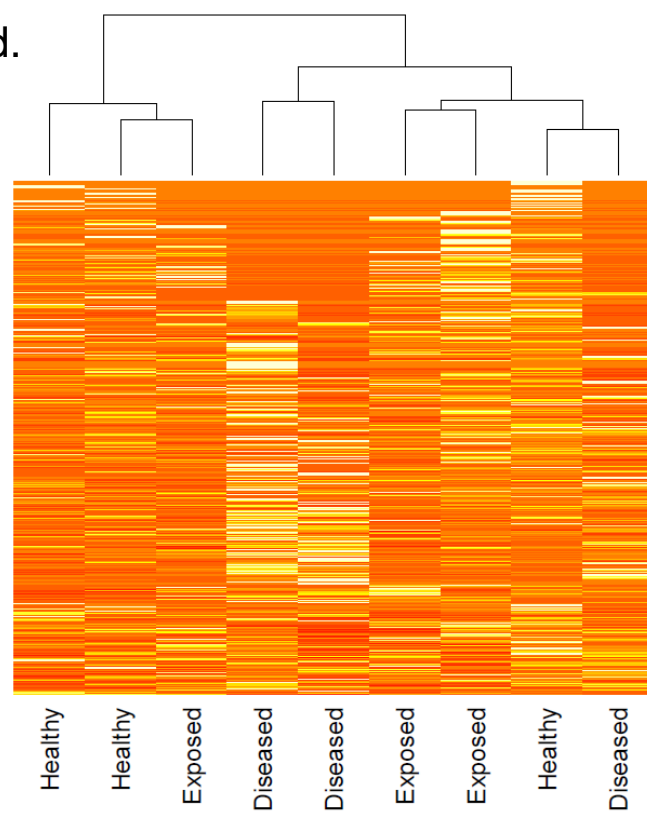

Supplement: S1 Fig — Heatmaps of the relative abundance of bacteria identified on each sample of healthy, exposed, and diseased coral tissue, tested in waterborne transmission experiments of dark-spot syndrome on Siderastrea siderea. Bacteria were examined at the class (a), order (b), family (c), and genus (d) taxonomic levels. Cluster dendrograms are indicated above each heatmap. (PDF) [file pone.0147493.s001.pdf]

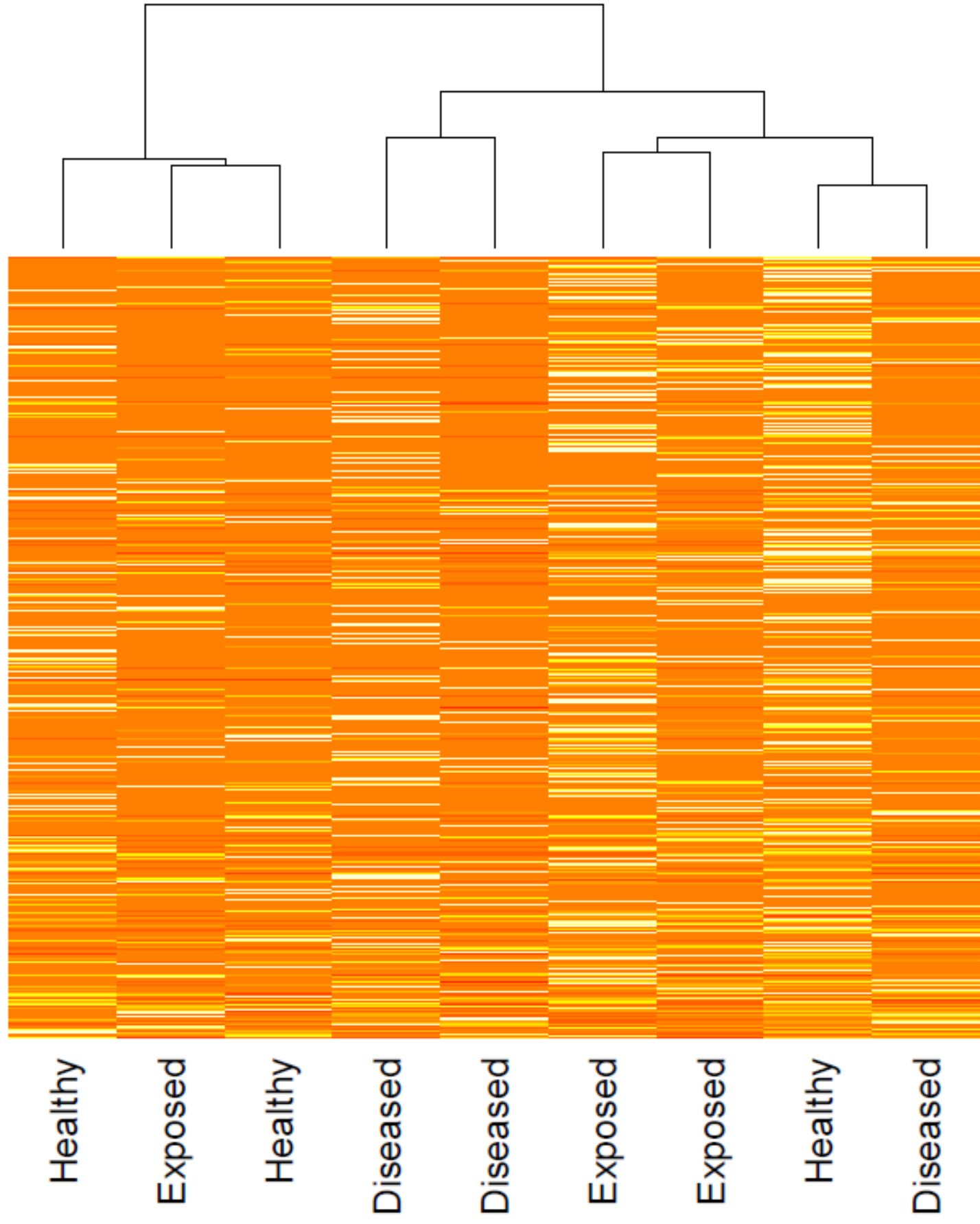

Supplement: S2 Fig — Heatmap of the relative abundance of operational taxonomic units (OTUs) identified on each sample of exposed, and diseased coral tissue, tested in waterborne transmission experiments of dark-spot syndrome on Siderastrea siderea. A cluster dendrogram is indicated above the heatmap. (PDF) [file pone.0147493.s002.pdf]

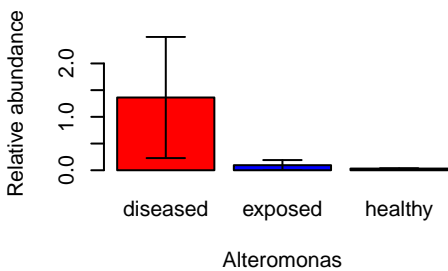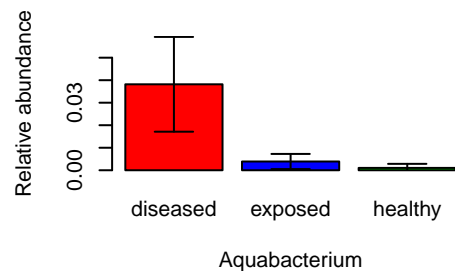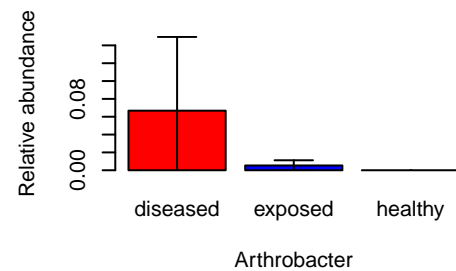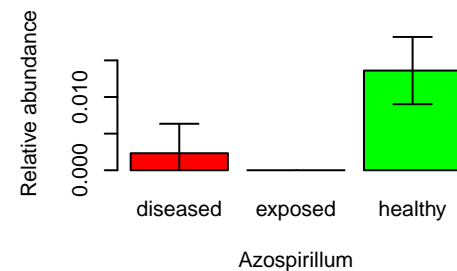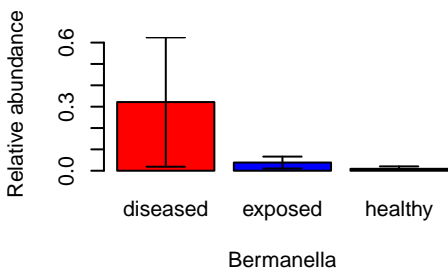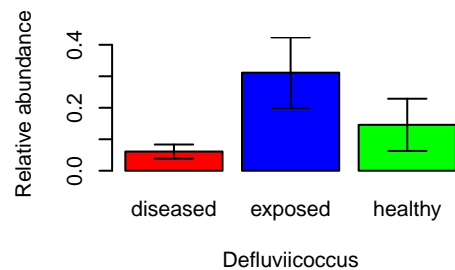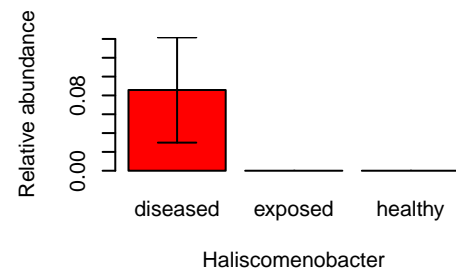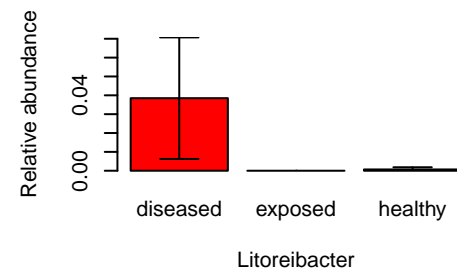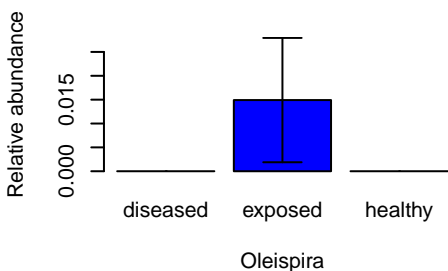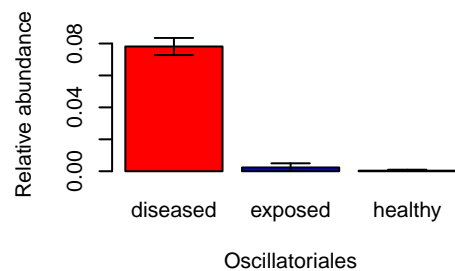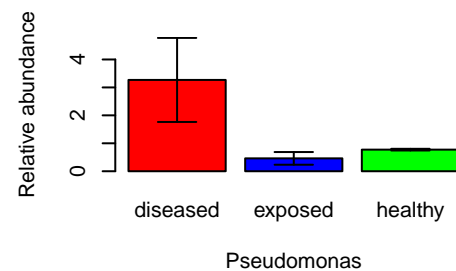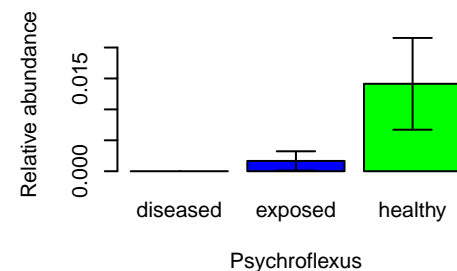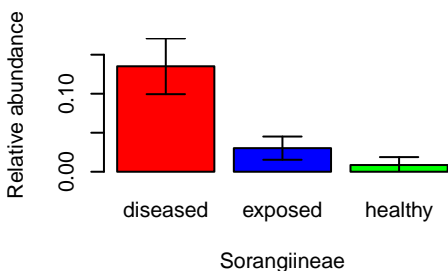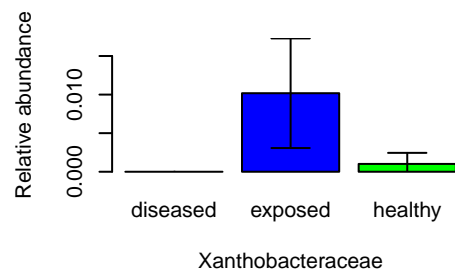

Supplement: S4 Fig — Only taxa whose relative abundances were significantly different among treatments, as determined by a Kruskal-Wallis rank-sum test using a chi-squared distribution are reported. Error bars denote standard deviation. n = 3. (PDF) [file pone.0147493.s004.pdf]

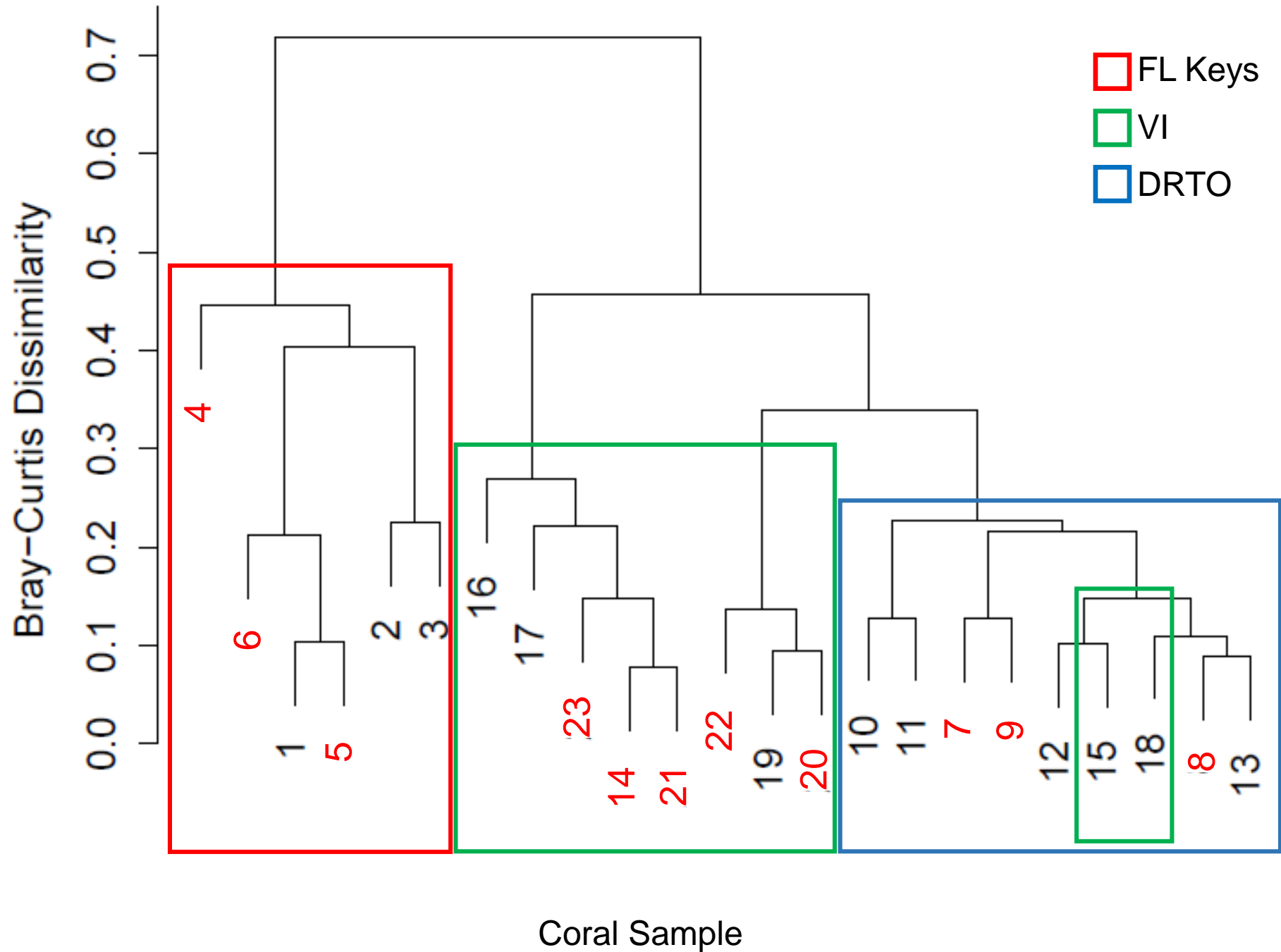

Supplement: S5 Fig — Cluster dendrogram is based on the relative abundance of class-level bacterial data from healthy and dark-spot affected Siderastrea siderea from the Florida Keys in 2013 (present study) compared with the Dry Tortugas and St. John, USVI in 2009 (Kellogg et al. [51]). Sample numbers in black represent apparently-healthy corals, whereas sample numbers in red represent corals with dark-spot syndrome. (PDF) [file pone.0147493.s005.pdf]
