# Supplementary material for: Does Dark-Spot Syndrome Experimentally Transmit among Caribbean Corals?
Source: PLoS One. 2016 Jan 20;11(1):e0147493. doi: 10.1371/journal.pone.0147493 (PMC4720368; doi:10.1371/journal.pone.0147493)
Supplement: S3 Fig — Shannon-Weiner diversity of microbes, at six taxonomic levels, in three samples each of healthy (H), exposed (E) and diseased (D) coral tissue, tested in waterborne transmission experiments of dark-spot syndrome on Siderastrea siderea. (PDF) [file pone.0147493.s003.pdf]

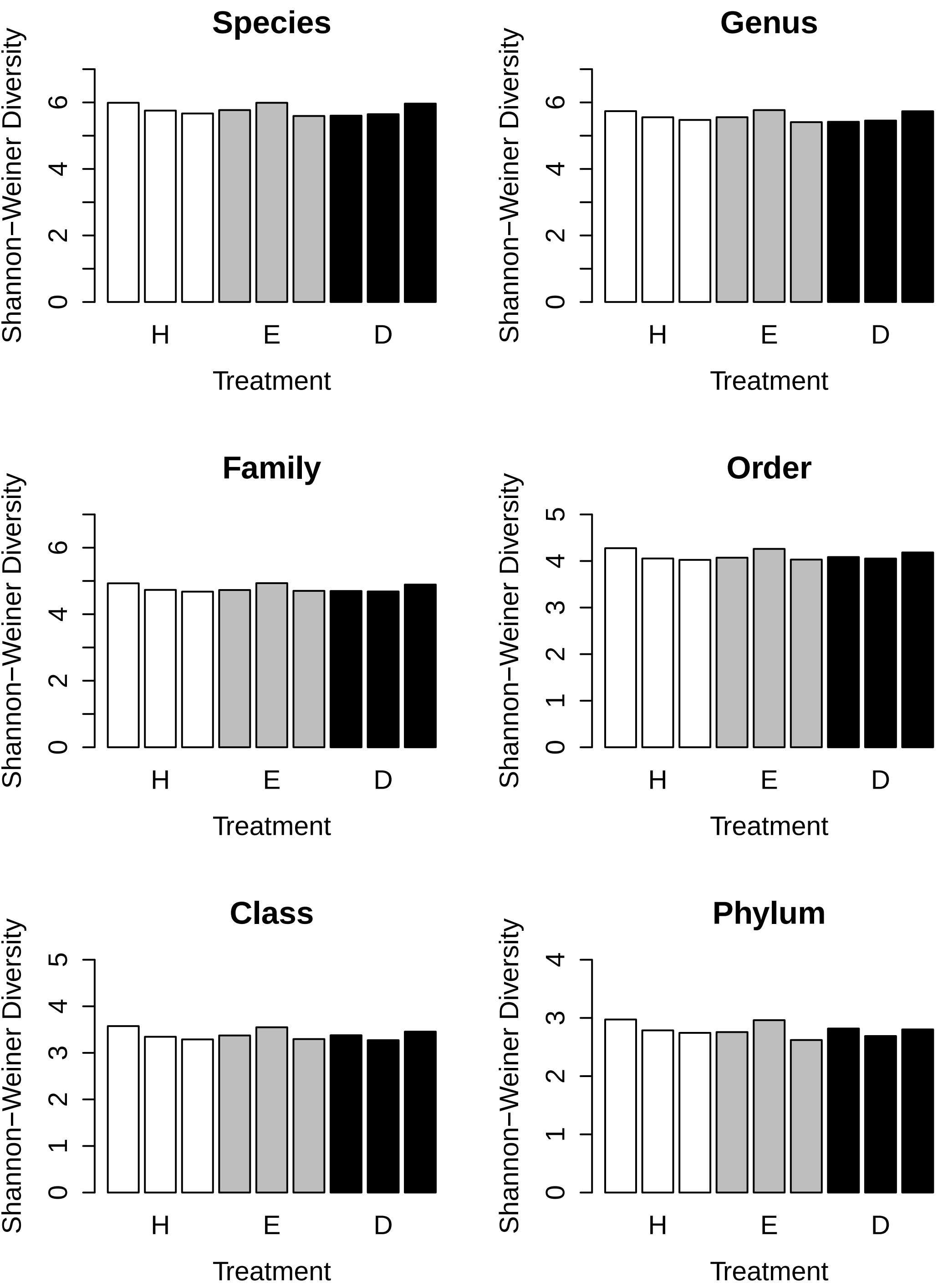
Species
Genus
Family
Order
Class
Phylum
Shannon−Weiner Diversity
Treatment
H
E
D
0
1
2
3
4
5
6
