# Supplementary material for: Does Dark-Spot Syndrome Experimentally Transmit among Caribbean Corals?
Source: PLoS One. 2016 Jan 20;11(1):e0147493. doi: 10.1371/journal.pone.0147493 (PMC4720368; doi:10.1371/journal.pone.0147493)
Supplement: S1 Table — Results of Kruskal-Wallis rank-sum tests using a chi-squared distribution comparing relative abundances of bacteria of six purported pathogenic taxa on diseased, exposed, and healthy corals colonies (~16 cm2) at the completion of laboratory-based dark-spot syndrome transmission experiments on Siderastrea siderea, where df = degrees of freedom, and n/a indicates that no Oscillatoria were identified in any sample. (DOCX) [file pone.0147493.s006.docx]

**S1 Table. Results of non-parametric analyses of variance of six purported pathogen taxa.**

| Taxa | p-value | df | Chi-sq |
| --- | --- | --- | --- |
| *Vibrio* | 0.49 | 2 | 1.42 |
| *Corynebacterium* | 0.94 | 2 | 0.13 |
| *Acinetobacter* | 0.73 | 2 | 0.62 |
| *Photobacterium* | 0.96 | 2 | 0.07 |
| *Parvularculaceae* | 0.73 | 2 | 0.62 |
| *Oscillatoria* | n/a | n/a | n/a |

Results of Kruskal-Wallis rank-sum tests using a chi-squared distribution comparing relative abundances of bacteria of six purported pathogenic taxa on diseased, exposed, and healthy corals colonies (~16 cm^2^) at the completion of laboratory-based dark-spot syndrome transmission experiments on *Siderastrea siderea,* where df = degrees of freedom, and n/a indicates that no *Oscillatoria* were identified in any sample.
